# Availability of behavioral health crisis care and associated changes in emergency department utilization

**DOI:** 10.1111/1475-6773.14368

**Published:** 2024-08-08

**Authors:** Ashlyn Burns, Joshua R. Vest, Nir Menachemi, Olena Mazurenko, Paul I. Musey, Michelle P. Salyers, Valerie A. Yeager

**Affiliations:** ^1^ Department of Health Policy and Management Indiana University Richard M. Fairbanks School of Public Health Indianapolis Indiana USA; ^2^ Regenstrief Institute Center for Biomedical Informatics Indiana University Richard M. Fairbanks School of Public Health Indianapolis Indiana USA; ^3^ Eskanazi Health Foundation Chair and Scholar of Emergency Medicine Indiana University School of Medicine Indianapolis Indiana USA; ^4^ Department of Psychology Indiana University School of Science Indianapolis Indiana USA

**Keywords:** behavioral health, crisis stabilization, emergency department utilization, emergency services, longitudinal panel

## Abstract

**Objective:**

To determine whether availability of behavioral health crisis care services is associated with changes in emergency department (ED) utilization.

**Data Sources and Study Setting:**

We used longitudinal panel data (2016–2021) on ED utilization from the Healthcare Cost and Utilization Project's State ED Databases and a novel dataset on crisis care services compiled using information from the Substance Abuse and Mental Health Services Administration's National Directories of Mental Health Treatment Facilities. A total of 1002 unique zip codes from Arizona, Florida, Kentucky, Maryland, and Wisconsin were included in our analyses.

**Study Design:**

To estimate the effect of crisis care availability on ED utilization, we used a linear regression model with zip code and year fixed effects and standard errors accounting for clustering at the zip code‐level. ED utilization related to mental, behavioral, and neurodevelopmental (MBD) disorders served as our primary outcome. We also examined pregnancy‐related ED utilization as a nonequivalent dependent variable to assess residual bias in effect estimates.

**Data Collection/Extraction Methods:**

We extracted data on crisis care services offered by mental health treatment facilities (*n* = 14,726 facility‐years) from the National Directories. MBD‐related ED utilization was assessed by applying the Clinical Classification Software Refined from the Healthcare Cost and Utilization Project to the primary ICD‐10‐CM diagnosis code on each ED encounter (*n* = 101,360,483). All data were aggregated to the zip code‐level (*n* = 6012 zip‐years).

**Principal Findings:**

The overall rate of MBD‐related ED visits between 2016 and 2021 was 1610 annual visits per 100,000 population. Walk‐in crisis stabilization services were associated with reduced MBD‐related ED utilization (coefficient = −0.028, *p* = 0.009), but were not significantly associated with changes in pregnancy‐related ED utilization.

**Conclusions:**

Walk‐in crisis stabilization services were associated with reductions in MBD‐related ED utilization. Decision‐makers looking to reduce MBD‐related ED utilization should consider increasing access to this promising alternative model.

**What is known on this topic**

- One in eight emergency department visits relates to a behavioral health condition, contributing to overcrowding and lengthy patient wait times that compromise patient quality of care.
- Behavioral health crisis care is a patient‐centered, cost‐effective alternative to the emergency department.
- However, existing evidence on the effectiveness of behavioral health crisis care for reducing emergency department utilization is limited primarily to case studies that do not measure actual emergency department utilization.

**What this study adds**

- We use a longitudinal panel analysis to examine population‐level changes in emergency department utilization associated with the availability of four components of behavioral health crisis care services offered by mental health treatment facilities.
- Our findings demonstrate that walk‐in crisis stabilization services are associated with reduced emergency department utilization related to mental, behavioral, and neurodevelopmental disorders.
- Increasing access to walk‐in crisis stabilization services appears to be an effective strategy for reducing emergency department utilization.

## 1 INTRODUCTION

With one in eight patients seeking care in the emergency department (ED) for a behavioral health crisis, behavioral health‐related ED visits account for a large portion of potentially preventable ED utilization.^1^ Behavioral health‐related utilization poses unique challenges for EDs, as a majority of EDs lack the space, personnel, and training needed to provide timely care and address the unique needs of patients with a behavioral health crisis.^2^ Importantly, behavioral health crises are increasing^1^, ^3^ at a much higher rate than overall ED utilization.^4^, ^5^ With the increase in anxiety and depression during the COVID‐19 pandemic, it is more important than ever to ensure access to a continuum of behavioral healthcare services, as untreated behavioral health conditions could lead to acute crises and more severe outcomes, such as suicide.^6^

Behavioral health crisis care (BHCC), an alternative care delivery model, offers a promising strategy to reduce ED overcrowding and an opportunity to increase access to more appropriate, specialized services.^3^, ^7^, ^8^ As defined by the Substance Abuse and Mental Health Services Administration (SAMHSA), BHCC includes emergency behavioral health services available on a 24/7 walk‐in basis to all patients, regardless of ability to pay.^3^ BHCC best practices include emergency psychiatric walk‐in services, crisis intervention teams, suicide prevention services, and peer support specialists.^3^ BHCC services may be offered by various organizations (e.g., hospitals, fire departments, etc.), but are most commonly delivered by mental health treatment facilities.^3^ These services may be delivered onsite, in the community where the individual is, or via telehealth.^3^ One goal of increasing access to BHCC is to reduce inappropriate ED utilization.^3^, ^9^ Treating patients with behavioral health needs in BHCC facilities instead of EDs may simultaneously lower healthcare costs while improving the quality of care patients receive as these facilities offer more specialized services appropriate for their unique needs. However, additional evidence is needed to quantify the relationship between BHCC availability and ED utilization.

The purpose of this study is to assess changes in ED utilization associated with availability of BHCC services. To learn more about this relationship, we linked data on mental health treatment facility locations and BHCC services from SAMHSA with data on ED utilization from the State Emergency Department Database (SEDD). Findings from this study will provide insight into the relationship between access to BHCC and ED utilization on a national level. As efforts are underway to develop comprehensive crisis response systems, this information may help inform decisions made by policy leaders, hospital managers, and behavioral health stakeholders.

## 2 METHODS

This study used a longitudinal panel analysis to examine the relationship between BHCC availability and ED utilization.

### 2.1 Data

We compiled a dataset on mental health treatment facility characteristics and availability of behavioral health services between 2016 and 2021 by utilizing SAMHSA's National Directories of Mental Health Treatment Facilities (hereafter, the National Directories). The National Directories are annual listings of federal, state, and local government facilities and private facilities that provide mental health treatment services and are publicly available at https://www.samhsa.gov/data/report/national-directory-mental-health-treatment-facilities. The National Directories include information on facilities that responded to the National Mental Health Services Survey (N‐MHSS), an annual survey conducted by SAMHSA. One member of the research team manually extracted information on facilities and services from the National Directories to create a longitudinal, facility‐level dataset (*n* = 14,726 facility‐years). The characteristics we extracted for each facility included ownership (private or public), setting (community mental health center, inpatient, outpatient, partial hospital, residential), payment methods (Medicare, Medicaid, private insurance, payment assistance, sliding fee scale), and services offered (crisis intervention teams, walk‐in crisis stabilization, suicide prevention services, peer support specialists).

We merged our dataset on BHCC services with data on ED utilization obtained from the Agency for Healthcare Research and Quality's (AHRQ) SEDD for five states: Arizona (AZ), Florida (FL), Kentucky (KY), Maryland (MD), and Wisconsin (WI). These data contain visit‐level information on ED visits (*n* = 101,360,483) that do not result in hospitalization, also known as “treat and release” encounters. The included states participated in the SEDD for all years between 2016 and 2021 and were purposively selected to ensure representativeness from different geographic regions of the United States and to ensure inclusion of states with BHCC services available. In general, when compared to other states, the selected states have a lower prevalence rate of behavioral health disorders and higher rates of access to care.^10^ Since we were interested in measuring changes in ED utilization at the population‐level, all facility‐ and visit‐level data were aggregated to the zip code‐level based on a list of zip codes obtained from the US Census Bureau.^11^ Consistent with previous research examining the influence of alternative care sites on ED utilization,^12^, ^13^ we used the patient's zip code reported in the SEDD to aggregate ED visits to the zip code‐level. The logic behind this approach is that most patients who seek emergency services do so near their home.^13^, ^14^, ^15^, ^16^, ^17^ Thus, when BHCC services are available within their home zip code, patients may be more likely to choose to visit these facilities rather than the ED. All zip codes within the five selected states with at least one ED visit in each year of the study period were included in our dataset (*n* = 18,354 zip‐years). Zip code‐level population size and 5‐year demographic estimates (2016–2021) were obtained from the Census data.

### 2.2 Missing data

One limitation we faced in extracting information to build our BHCC dataset was that SAMHSA does not provide a unique facility identifier in the National Directories that can be used to link facility‐level data across years. In building our dataset, we used the facility address listed in the National Directories to assign facility numbers and link data on individual facilities across years. If a facility's name changed, but the address remained the same, we assumed that the facility likely experienced a change in ownership or rebranded but considered this as the same facility in our dataset. However, if a facility was not represented in a given year, it was impossible for us to tell from the National Directories alone whether a facility was closed at the time or simply did not respond to the N‐MHSS that year. When a facility was missing from the directory in 1 year but reported services in both the prior year and following year, we assumed the facility did not report services and replaced the missing value with the value from the prior year. If a facility did not report for two or more years in a row, we assumed the facility closed.

### 2.3 Independent variables

This study's primary independent variables of interest were four indicators representing BHCC services (crisis intervention teams, walk‐in crisis stabilization, suicide prevention services, peer support specialists). Our dataset included an indicator for whether these services were offered each year, allowing us to identify when a facility started or stopped offering a service within the study period. We aggregated these indicators to the zip code‐level to create a count representing the number of mental health treatment facilities in the zip code offering each service. We also aggregated facility characteristics to the zip code‐level to control for the total number of mental health treatment facilities in the zip code, the percentage of facilities that were publicly versus privately owned, the percentage of facilities accepting various payment methods (Medicare, Medicaid, private insurance, payment assistance, sliding fee scale), and the percentage of facilities providing different settings of care (community mental health center, inpatient, outpatient, partial hospital, residential).

### 2.4 Dependent variables

ED utilization rates were our primary variables of interest. Given that we would expect BHCC services to reduce ED visits related to behavioral health crises, but not ED visits for other health needs, we created an indicator for the primary visit reason using the Clinical Classification Software Refined (CCSR) for ICD‐10‐CM from AHRQ's Healthcare Cost and Utilization Project.^18^ The CCSR aggregates ICD‐10‐CM diagnosis codes into clinically meaningful categories across 22 body systems, providing a means to identify specific clinical conditions.^18^ The body system category for mental, behavioral, and neurodevelopmental disorders (“MBD”) contains 32 categories, including depressive disorders, alcohol‐related disorders, and suicidal ideation/self‐harm.^19^ We applied the CCSR to the primary ICD‐10‐CM diagnosis code on each ED encounter in the SEDD dataset to distinguish between MBD‐related and non‐MBD‐related ED visits. In doing so, we derived a binary variable indicating whether the encounter had an MBD diagnosis as the primary visit reason. To examine population‐level changes in MBD‐related ED utilization, we aggregated this indicator to the zip‐code level, generating the total number of MBD‐related ED visits per zip code. To adjust for population size, we converted the total number of MBD‐related ED visits to a rate per 100,000 population. Finally, as the distribution of this outcome variable was skewed, we log‐transformed the rate of ED visits per 100,000, approximating a normal distribution and minimizing the influence of outliers.

In addition to our primary outcome variable, we also constructed a control outcome. Control outcomes improve internal validity by assessing residual bias in effect estimates.^20^ We used pregnancy‐related ED utilization as a control outcome, as we do not expect access to BHCC services to influence ED utilization for pregnancy‐related conditions. Following a similar process, we applied the CCSR to the primary ICD‐10‐CM diagnosis code on each ED encounter in the SEDD dataset to distinguish between pregnancy‐related and non‐pregnancy‐related ED visits. In doing so, we derived a binary variable indicating whether the encounter had a pregnancy‐related diagnosis as the primary visit reason. We then followed the same process to aggregate this indicator to the zip‐code level, convert the total number of pregnancy‐related ED visits to a rate per 100,000 population, and log‐transformed this outcome.

### 2.5 Analyses

We used longitudinal panel data to examine the relationship between BHCC availability over time and the rate of MBD‐related ED utilization at the zip code‐level. To estimate the effect of BHCC availability on MBD‐related ED utilization, we used a linear regression model with a two‐way fixed effects approach, where the independent variable is indexed to zip code z in year t, and standard errors accounting for clustering at the zip code‐level. Zip code fixed effects control for time‐invariant unobserved zip code attributes that may affect MBD‐related ED utilization at the zip code‐level, whereas year fixed effects control for time trends.

For our primary analyses, we limited our sample to zip codes with at least one mental health treatment facility (*n* = 6012). We excluded zip codes without a mental health treatment facility at all because these communities do not have BHCC services by definition, and thus are not the best comparison group for communities with mental health treatment facilities that have adopted BHCC services. In addition to our primary analyses, we conducted several sensitivity analyses. First, we repeated our main regression model with all zip codes from the five selected states, including those without a mental health treatment facility, in the sample (*n* = 18,354 zip‐years). Second, given the influence of the COVID‐19 pandemic on ED utilization, we excluded 2020 and 2021 and repeated our main regression model limited to the years 2016–2019 (*n* = 4008 zip‐years). Finally, we stratified by urban (*n* = 4494 zip‐years) and rural (*n* = 1518 zip‐years) zip codes to assess potential differences by geographic type. Rural and urban zip codes were classified using the Rural Urban Commuting Area Codes from the USDA (1–3 = urban, 4–10 = rural).^21^ The Institutional Review Board at Indiana University determined this study was not human subjects research because all data were de‐identified and no human subjects were directly involved in the study.

## 3 RESULTS

A total of 1002 unique zip codes (*n* = 6012 zip‐years) from AZ, FL, KY, MD, and WI were included in our primary analyses. Table 1 shows zip code‐level summary statistics. The overall rate of MBD‐related ED visits reported from included zip codes between 2016 and 2021 was 1610 visits per 100,000 population. On average, zip codes had one mental health treatment facility offering suicide prevention services and less than one facility offering the following services: crisis intervention teams, walk‐in crisis stabilization, and peer support specialists. The average total number of mental health treatment facilities per zip code was 2.5.

**TABLE 1:** Zip code‐level summary statistics (*n* = 6012 zip‐years).

Summary statistics	Mean	(SD)
Facilities with behavioral health crisis care services		
Walk‐in crisis stabilization	0.49	(0.73)
Peer support specialists	0.46	(0.80)
Crisis intervention teams	0.69	(0.91)
Suicide prevention	1.04	(1.17)
Facility characteristics		
Total mental health treatment facilities	2.45	(2.15)
Percent publicly owned	63.24	(39.02)
Facility setting		
Percent outpatient	58.09	(39.84)
Percent community mental health center	13.88	(29.76)
Percent residential	10.77	(25.76)
Percent partial hospital	9.08	(23.11)
Percent inpatient	8.40	(22.03)
Payment methods accepted		
Percent accepting Medicaid	62.08	(39.18)
Percent accepting private insurance	58.80	(40.16)
Percent accepting Medicare	47.30	(40.76)
Percent with sliding pay scales	26.84	(36.87)
Percent providing payment assistance	12.67	(27.71)
Emergency department utilization		
MBD‐related ED visits per 100,000	1610	(4462)
Pregnancy‐related ED visits per 100,000	1183	(905)

*Note*: MBD refers to a body systems category containing diagnoses related to mental, behavioral, and neurodevelopmental disorders, as defined by the Clinical Classification Software Refined (CCSR).^18^

Table 2 shows more detailed information on zip code‐level BHCC availability between 2016 and 2021. While some zip codes started out without BHCC services and gained them during the study period, other zip codes did not have BHCC services available in any years of the study. Availability of BHCC services fluctuated throughout the study period, with availability of some services increasing 1 year and decreasing the next. For example, the number of facilities offering crisis intervention teams, walk‐in services, and suicide prevention services decreased from 2020 to 2021, whereas peer support specialists increased. In 2021, 362 of the zip codes examined (36.1%) had at least one mental health treatment facility that offered walk‐in crisis stabilization services, 425 (42.4%) had a facility offering peer support services, 473 (47.2%) had a facility offering crisis intervention teams, and 699 (69.8%) had a facility offering suicide prevention services.

**TABLE 2:** Zip code‐level behavioral health crisis care availability by year, 2016–2021 (*n* = 6012 zip‐years).

Service type	2016		2017		2018		2019		2020		2021	
	*n*	%	*n*	%	*n*	%	*n*	%	*n*	%	*n*	%
Walk‐in crisis stabilization	373	37.2	394	39.3	388	38.7	400	39.9	412	41.1	362	36.1
Peer support specialists	281	28.0	307	30.6	298	29.7	352	35.1	418	41.7	425	42.4
Crisis intervention teams	486	48.5	511	51.0	496	49.5	515	51.4	514	51.3	473	47.2
Suicide prevention	586	58.5	639	63.8	620	61.9	678	67.7	702	70.1	699	69.8
Total facilities	2456	‐	2455	‐	2455	‐	2454	‐	2454	‐	2452	‐

*Note*: Zip code‐level availability represents the number of zip codes with at least one mental health treatment facility offering the listed service in a given year (*n* = 1002 unique zip codes per year). Total facilities represent the total number of open mental health treatment facilities based on information obtained from the Substance Abuse and Mental Health Services Administration's National Directories of Mental Health Treatment Facilities.

Results from linear fixed effects regression analysis predicting the log‐transformed rate of MBD‐related ED utilization per 100,000 are presented in Table 3. In our initial analyses examining walk‐in crisis stabilization and year only (Model 1), availability of walk‐in BHCC services was associated with reduced MBD‐related ED utilization (coefficient = −0.020, *p* = 0.036). This finding remained consistent when controlling for other BHCC services (Model 2; coefficient = −0.028, *p* = 0.009) and when controlling for additional zip‐code level facility characteristics (Model 3; coefficient = −0.027, *p* = 0.015). Availability of crisis intervention teams, suicide prevention services, and peer support specialists were not significantly associated with changes in MBD‐related ED utilization in any models.

**TABLE 3:** Effect of adoption of behavioral health crisis care (BHCC) services on mental, behavioral, and neurodevelopmental (MBD)‐related emergency department utilization, 2016–2021.

Organizational characteristics	Model 1				Model 2				Model 3			
	Coef.	*p*‐value	95% CI		Coef.	*p*‐value	95% CI		Coef.	*p*‐value	95% CI	
BHCC services												
Walk‐in crisis stabilization	**−0.020**	**0.036**	**−0.038**	**−0.001**	**−0.028**	**0.009**	**−0.049**	**−0.007**	**−0.027**	**0.015**	**−0.048**	**−0.005**
Crisis intervention teams	‐	‐	‐	‐	0.010	0.168	−0.004	0.023	0.009	0.205	−0.005	0.023
Suicide prevention	‐	‐	‐	‐	0.007	0.237	−0.005	0.020	0.007	0.266	−0.006	0.020
Peer support specialists	‐	‐	‐	‐	−0.001	0.975	−0.015	0.015	−0.001	0.946	−0.016	0.015
Year (reference = 2016)												
2017	−0.011	0.245	−0.029	0.008	−0.012	0.207	−0.031	0.007	−0.013	0.195	−0.032	0.006
2018	0.009	0.163	−0.004	0.022	0.008	0.225	−0.005	0.022	0.008	0.249	−0.006	0.021
2019	**0.020**	**0.012**	**0.005**	**0.036**	**0.018**	**0.034**	**0.001**	**0.035**	**0.018**	**0.041**	**0.001**	**0.034**
2020	**−0.091**	**<0.001**	**−0.108**	**−0.074**	**−0.094**	**<0.001**	**−0.112**	**−0.076**	**−0.095**	**<0.001**	**−0.113**	**−0.076**
2021	**−0.021**	**0.015**	**−0.038**	**−0.004**	**−0.024**	**0.013**	**−0.042**	**−0.005**	**−0.025**	**0.01**	**−0.044**	**−0.006**
Zip code‐level facility characteristics												
Total mental health treatment facilities	‐	‐			‐	‐			−0.002	0.954	−0.055	0.052
Percent publicly owned					‐	‐			0.001	0.155	−0.001	0.001
Percent outpatient	‐	‐			‐	‐			−0.001	0.703	−0.001	0.001
Percent community mental health center	‐	‐			‐	‐			−0.001	0.357	−0.001	0.001
Percent residential	‐	‐			‐	‐			0.001	0.417	−0.001	0.001
Percent partial hospital	‐	‐			‐	‐			−0.001	0.580	−0.001	0.001
Percent inpatient	‐	‐			‐	‐			0.001	0.933	−0.001	0.001
Percent accepting Medicaid	‐	‐			‐	‐			0.001	0.934	−0.001	0.001
Percent accepting private insurance	‐	‐			‐	‐			−0.001	0.162	−0.001	0.001
Percent accepting Medicare	‐	‐			‐	‐			−0.001	0.747	−0.001	0.001
Percent with sliding pay scales	‐	‐			‐	‐			−0.001	0.426	−0.001	0.001
Percent providing payment assistance	‐	‐			‐	‐			0.001	0.253	−0.001	0.001

*Note*: Bold text indicates statistically significant finding. Model 1 examines the relationship between availability of walk‐in crisis stabilization services and MBD‐related ED utilization while control for year only. Model 2 controls for availability of additional BHCC services. Model 3 controls for the characteristics/service types of all mental health treatment facilities in the zip code.

Findings from sensitivity analyses are presented in Table 4. When including all zip codes in the five selected states (Model 4), availability of walk‐in crisis stabilization services remained significantly associated with reduced MBD‐related ED utilization (coefficient = −0.026, *p* = 0.017). This finding also remained consistent when the years 2020 and 2021 were excluded from analyses (Model 5; coefficient = −0.031, *p* = 0.023). When limited to urban zip codes only (Model 6), availability of walk‐in crisis stabilization was no longer significant at the *p* < 0.05 level, but the direction of the coefficient remained consistent (coefficient = −0.017, *p* = 0.075). When limited to rural zip codes (Model 7), availability of walk‐in crisis stabilization services remained significantly associated with reduced MBD‐related ED utilization (coefficient = −0.069, *p* = 0.025). No other services were significantly associated with changes in MBD‐related ED utilization in the sensitivity analyses.

**TABLE 4:** Effect of adoption of behavioral health crisis care (BHCC) services on mental, behavioral, and neurodevelopmental‐related emergency department utilization, sensitivity analyses, 2016–2021.

Organizational characteristics	Model 4				Model 5				Model 6				Model 7			
	Coef.	*p*‐value	95% CI		Coef.	*p*‐value	95% CI		Coef.	*p*‐value	95% CI		Coef.	*p*‐value	95% CI	
BHCC services																
Walk‐in crisis stabilization	**−0.026**	**0.017**	**−0.047**	**−0.005**	**−0.031**	**0.023**	**−0.058**	**−0.004**	−0.017	0.075	**−0.035**	**0.002**	**−0.069**	**0.025**	**−0.130**	**−0.009**
Crisis intervention teams	0.011	0.113	−0.003	0.025	−0.002	0.853	−0.024	0.020	0.012	0.062	−0.001	0.025	−0.011	0.682	−0.063	0.041
Suicide prevention	0.005	0.407	−0.007	0.017	0.009	0.268	−0.007	0.026	0.006	0.280	−0.005	0.018	0.023	0.239	−0.015	0.061
Peer support specialists	−0.003	0.704	−0.018	0.012	0.008	0.482	−0.016	0.034	−0.003	0.744	−0.018	0.013	−0.013	0.564	−0.031	0.056
Year (reference = 2016)																
2017	−0.013	0.101	−0.029	0.003	−0.012	0.222	−0.031	0.007	−0.009	0.354	−0.027	0.010	−0.026	0.336	−0.079	0.027
2018	**0.017**	**0.015**	0.004	0.032	0.008	0.233	−0.005	0.022	0.011	0.103	−0.002	0.024	−0.002	0.913	−0.038	0.034
2019	**0.031**	**<0.001**	**0.016**	**0.046**	**0.018**	**0.044**	**0.001**	**0.035**	**0.021**	**0.008**	0.006	0.037	0.007	0.761	−0.039	0.054
2020	**−0.089**	**<0.001**	−0.105	−0.073	‐	‐	‐	‐	**−0.103**	**<0.001**	**−0.121**	**−0.084**	**−0.070**	**0.004**	**−0.117**	**−0.022**
2021	−0.001	0.838	−0.018	0.015	‐	‐	‐	‐	**−0.033**	**0.002**	−0.053	−0.012	0.001	0.946	−0.042	0.045

*Note*: Bold text indicates statistically significant finding. Model 4 includes zip codes without mental health treatment facilities in the five selected states (*n* = 18,354 zip‐years). Model 5 includes only zip codes with mental health treatment facilities in years 2016–2019 (*n* = 4008 zip‐years). Model 6 includes urban zip codes with mental health treatment facilities only (*n* = 4494 zip‐years). Model 7 includes rural zip codes only (*n* = 1518 zip‐years). Zip codes were designated as urban versus rural using the Rural Urban Commuting Area Codes from the USDA (codes 1–3 = urban, 4–10 = rural).

Results from linear fixed effects regression analysis predicting the log‐transformed rate of pregnancy‐related ED utilization per 100,000 are presented in Table 5. Availability of walk‐in BHCC services was not significantly associated with change in pregnancy‐related ED utilization in any model. This finding also remained consistent across all sensitivity analyses (not shown).

**TABLE 5:** Effect of adoption of behavioral health crisis care (BHCC) services on pregnancy‐related emergency department utilization, 2016–2021.

Organizational characteristics	Model 1				Model 2				Model 3			
	Coef.	*p*‐value	95% CI		Coef.	*p*‐value	95% CI		Coef.	*p*‐value	95% CI	
BHCC services												
Walk‐in crisis stabilization	0.002	0.817	−0.015	0.019	0.003	0.760	−0.017	0.023	0.004	0.734	−0.017	0.024
Crisis intervention teams	‐	‐	‐	‐	0.004	0.587	−0.010	0.018	0.003	0.702	−0.012	0.018
Suicide prevention	‐	‐	‐	‐	−0.007	0.313	−0.019	0.006	−0.005	0.461	−0.018	0.008
Peer support specialists	‐	‐	‐	‐	0.003	0.683	−0.011	0.018	0.004	0.618	−0.011	0.019
Year (reference = 2016)												
2017	0.017	0.106	−0.004	0.039	0.018	0.100	−0.003	0.040	0.018	0.106	−0.004	0.040
2018	**0.060**	**<0.001**	**0.042**	**0.078**	**0.061**	**<0.001**	**0.043**	**0.079**	**0.060**	**<0.001**	**0.041**	**0.078**
2019	**0.060**	**<0.001**	**0.041**	**0.079**	**0.061**	**<0.001**	**0.042**	**0.081**	**0.059**	**<0.001**	**0.039**	**0.078**
2020	**−0.144**	**<0.001**	**−0.164**	**−0.124**	**−0.142**	**<0.001**	**−0.163**	**−0.121**	**−0.145**	**<0.001**	**−0.166**	**−0.123**
2021	−0.006	0.589	−0.029	0.016	−0.005	0.692	−0.029	0.019	−0.008	0.521	−0.032	0.016
Zip code‐level facility characteristics												
Total mental health treatment facilities	‐	‐			‐	‐			−0.006	0.871	−0.084	0.071
Percent publicly owned					‐	‐			0.001	0.747	−0.001	0.001
Percent outpatient	‐	‐			‐	‐			−0.001	0.913	−0.001	0.001
Percent community mental health center	‐	‐			‐	‐			**−0.001**	**0.043**	**−0.001**	**−0.001**
Percent residential	‐	‐			‐	‐			0.001	0.292	−0.001	0.001
Percent partial hospital	‐	‐			‐	‐			−0.001	0.820	−0.001	0.001
Percent inpatient	‐	‐			‐	‐			0.001	0.601	−0.001	0.001
Percent accepting Medicaid	‐	‐			‐	‐			0.001	0.675	−0.001	0.001
Percent accepting private insurance	‐	‐			‐	‐			**0.001**	**0.026**	**0.001**	**0.001**
Percent accepting Medicare	‐	‐			‐	‐			−0.001	0.102	−0.001	0.001
Percent with sliding pay scales	‐	‐			‐	‐			−0.001	0.274	−0.001	0.001
Percent providing payment assistance	‐	‐			‐	‐			**0.001**	**0.028**	**0.001**	**0.001**

*Note*: Bold text indicates statistically significant finding. Pregnancy‐related ED utilization was examined as a control outcome. Model 1 examines the relationship between availability of walk‐in crisis stabilization services and pregnancy‐related ED utilization while controlling for year. Model 2 controls for availability of additional BHCC services. Model 3 controls for the characteristics/service types of all mental health treatment facilities in the zip code.

## 4 DISCUSSION

ED utilization for behavioral health crises is costly and inefficient for both hospitals and patients, necessitating alternative strategies for delivering care to individuals in crisis. This study builds on prior case studies on alternate models for delivering emergency behavioral health services^8^, ^22^, ^23^, ^24^ by examining changes in MBD‐related ED utilization at the population‐level across a longitudinal panel. We examined changes in MBD‐related ED utilization associated with BHCC services. We found that availability of walk‐in BHCC services was associated with reduced MBD‐related ED utilization. More specifically, for each additional mental health treatment facility that offered walk‐in crisis stabilization services, we saw a 2.8% decrease in the mean rate of MBD‐related ED utilization (based on the coefficient from Model 2). Our confidence in this finding was strengthened by a null finding when examining the relationship between availability of walk‐in BHCC services and pregnancy‐related ED utilization as a control outcome. When communities have another site that serves walk‐in patients with urgent needs, it is possible that individuals may turn to these facilities for care instead of the ED. Overall, findings from this study provide additional support for claims that increasing access to BHCC services is a potential strategy for diverting individuals from the ED, highlighting the importance of access to walk‐in BHCC services.

Similar to EDs, mental health treatment facilities that accept walk‐ins provide individuals in crisis a place where they can get help when they need it, without having to wait for an appointment.^3^ Walk‐in access is important for meeting the needs of patients in crisis, which require urgent attention. Notably, while we found that availability of walk‐in BHCC services by mental health treatment facilities was associated with reduced MBD‐related ED utilization among the zip code population, we also found that many zip codes lack access to this service. In fact, we saw a 16% decline in the number of facilities offering walk‐in services from 2020 to 2021, overlapping with the onset of the COVID‐19 pandemic. Previous studies of national availability of walk‐in crisis services reported a 16% decrease in services from 2014 to 2018,^25^, ^26^, ^27^ suggesting that service availability was already declining and took an even steeper drop in 2021. The continued decline in availability of these services is concerning, especially given our findings suggesting that these services have the potential to reduce ED utilization. As efforts to establish a national crisis system are underway, policies are needed to address disparities and ensure equitable access to both routine behavioral health services and crisis services. For example, recent efforts to transform the delivery of behavioral health services by expanding Certified Community Behavioral Health Clinics (CCBHCs) across the United States provide a promising opportunity to address gaps in access to care by increasing availability of both routine services for crisis prevention and BHCC services for acute crises that require urgent intervention.^28^

While availability of walk‐in crisis stabilization services was no longer significantly related to changes in MBD‐related ED utilization in urban communities when stratifying by urban/rural zip codes, this relationship remained significant and had a much stronger effect size in rural zip codes. Given documented disparities in access to BHCC services in rural communities,^29^, ^30^ increasing availability of walk‐in crisis stabilization services may be particularly impactful in rural communities, where individuals have fewer options for places to seek care. As for urban communities, given their large population size, one possible explanation for the lack of an effect when limited to urban communities is that BHCC services may not have enough capacity to result in a statistically meaningful change in population‐level ED utilization. In the current study, we were able to measure the number of facilities offering each BHCC service in the zip code, but we were not able to account for the capacity of services at individual locations (e.g., the number of beds available for stabilization services, the number of full‐time equivalent employees dedicated to each service type, etc.). Future research should examine more detailed measures of capacity, as well as other types of BHCC services (e.g., mobile crisis services, telehealth services) not examined in the present study.

This study has strengths and weaknesses. Using the National Directories allowed us to identify the exact location of facilities and BHCC services offered in a given year, facilitating our ability to conduct more granular analyses than previous studies using N‐MHSS data. However, because some facilities that complete the N‐MHSS opt out of inclusion in the National Directories, such facilities are not represented in our dataset. While examining BHCC availability and ED utilization at the zip code level provides deeper insight than previous studies, the use of zip codes is also a limitation as this geographic unit may not represent actual service areas and some patients may seek care outside of the zip code where they live. It is possible that zip codes are not the best geographic unit for analyses but given that we do not know the actual service areas of the facilities in our sample, zip codes served as the most reasonable geography we could measure. It is also possible that some facilities may have offered services that were not reflected in reporting, particularly if they did not respond to the N‐MHSS in a given year. Additionally, we were able to control for the types of insurance accepted by mental health treatment facilities in our sample but did not have detailed information about payer mix or reimbursement rates for these organizations, which may be of interest in future studies. Furthermore, we relied on the primary admission diagnosis on the ED visit record to determine whether the visit was MBD‐related or not. However, it is possible that patients experiencing a behavioral health crisis may have initially presented with another symptom (e.g., chest pain, trouble breathing) related to anxiety that was recorded by the physician under a different diagnostic code. This may have influenced overall rates of MBD‐related ED utilization, introducing conservative bias. Finally, our study period overlaps with the COVID‐19 pandemic, which influenced overall ED utilization and may have influenced our findings.

## 5 CONCLUSIONS

We found that availability of walk‐in crisis stabilization services was associated with reduced ED utilization for behavioral health crises. When people have another place that they can go to, or another way to reach out for help, this provides them with an alternative to the ED. These findings provide evidence supporting claims that walk‐in crisis stabilization services offered by mental health treatment facilities are a promising alternative model for diverting patients experiencing a behavioral health crisis from the ED. Yet, availability of these services remains limited. As changes to the national crisis care delivery system are underway, ensuring access to walk‐in crisis stabilization services is critical to preventing ED utilization and delivering patient‐centered care.
